# Use of nPSi-βCD Composite Microparticles for the Controlled Release of Caffeic Acid and Pinocembrin, Two Main Polyphenolic Compounds Found in a Chilean Propolis

**DOI:** 10.3390/pharmaceutics11060289

**Published:** 2019-06-19

**Authors:** Dina Guzmán-Oyarzo, Tanya Plaza, Gonzalo Recio-Sánchez, Dulcineia S. P. Abdalla, Luis A. Salazar, Jacobo Hernández-Montelongo

**Affiliations:** 1 Center of Molecular Biology and Pharmacogenetics, Scientific and Technological Bioresource Nucleus (BIOREN), Universidad de La Frontera, Avenida Francisco Salazar 01145, Temuco 4811230, Chile; dina.guzman.o@gmail.com; 2 Bioproducts and Advanced Materials Research Center (BioMA), Faculty of Engineering, Universidad Católica de Temuco, Avenida Rudecindo Ortega 02950, Temuco 4813302, Chile; tplaza.neira@gmail.com (T.P.); grecio@uct.cl (G.R.-S.); 3 Department of Physical and Mathematical Sciences, Faculty of Engineering, Universidad Católica de Temuco, Temuco 4813302, Chile; 4 Department of Clinical and Toxicological Analyses, Faculty of Pharmaceutical Sciences, Universidade de São Paulo, Avenida Professor Lineu Prestes 580, CEP 05508-000 São Paulo, SP, Brazil; dspabdalla@gmail.com

**Keywords:** controlled release, nanoporous silicon, βCD polymer, caffeic acid, pinocembrin, polyphenols, HUVECs

## Abstract

Propolis is widely recognized for its various therapeutic properties. These are attributed to its rich composition in polyphenols, which exhibit multiple biological properties (e.g., antioxidant, anti-inflammatory, anti-angiogenic). Despite its multiple benefits, oral administration of polyphenols results in low bioavailability at the action site. An alternative to face this problem is the use of biomaterials at nano-micro scale due to its high versatility as carriers and delivery systems of various drugs and biomolecules. The aim of this work is to determine if nPSi-βCD microparticles are a suitable material for the load and controlled release of caffeic acid (CA) and pinocembrin (Pin), two of the main components of a Chilean propolis with anti-atherogenic and anti-angiogenic activity. Polyphenols and nPSi-βCD microparticles cytocompatibility studies were carried out with human umbilical vein endothelial cells (HUVECs). Results from physicochemical characterization demonstrated nPSi-βCD microparticles successfully retained and controlled release CA and Pin. Furthermore, nPSi-βCD microparticles presented cytocompatibility with HUVECs culture at concentrations of 0.25 mg/mL. These results suggest that nPSi-βCD microparticles could safely be used as an alternate oral delivery system to improve controlled release and bioavailability of CA or Pin—and eventually other polyphenols—thus enhancing its therapeutic effect for the treatment of different diseases.

## 1. Introduction

Since ancient times, the use of natural compounds has been of great importance for medicine mainly in the prevention and treatment of different pathologies [1,2]. That is why they represent the main source of used compounds in the discovery and/or development of new drugs [3]. An example of natural compounds with bioactive potential is propolis, which is a resinous compound produced by bees from plants exudates. Studies both in vitro and in vivo have identified a wide variety of biological activities for propolis: antibacterial [4], antifungal [5], antioxidant [6], anti-inflammatory [7], anti-carcinogenic [8] and anti-angiogenic [9]. These activities are attributed to its polyphenols rich composition, molecules that present different biological properties: relaxing [10], antioxidant [11], antithrombotic [12], antiangiogenic [13], anti-inflammatory [14], anti-carcinogenic [15], among others. Biochemically, polyphenols are secondary metabolites exclusively synthesized by plants and their entire structure is based in one or more hydroxyl groups attached to an aromatic ring (benzene) [16]. Since the role of polyphenols in plants is related to growth, development and defense, they are found in leaves, fruits and seeds, as well as in a wide range of food of plant origin (vegetables, tea, cocoa, wine, etc.) [17]. Concerning to the presence and abundance of polyphenols in propolis, they are very variable due to their close dependence with the botanical origin of plants, climate, geographical location, year and time of collection [3,18,19]. Examples of this important dependence are the studies of three Brazilians, one Polish and one Chilean propolis. For the Brazilians propolis, Daleprane et al. [9] reported that artepellin C, pinocembrin and kampferol were the main components of green propolis; 3-hydroxy-8,9-dimethoxypterocarpane, medicarpine and daidezein were the main components of red propolis; and pinocembrin, phenyl ester of caffeic acid, quercetin and galangin were the main components of brown propolis. For the Polish propolis, Szliszka et al. [20] detected that was mainly composed by the flavonoids pinobanksin, chrysin and methoxyflavanone; and the phenolics acids coumarin, ferulic and caffeic. Finally, for the Chilean propolis with anti-atherogenic and anti-angiogenic activity [21], the main polyphenols detected in the ethanolic extract were caffeic acid (a phenolic acid) and pinocembrin (a flavonone) [22].

Daily intake of polyphenols has multiple health benefits [23] because they reduce the risk of developing non-communicable diseases such as diabetes [11], cancer [24] and cardiovascular diseases [25]. In vivo studies reported that supplementation of the diet with persimmon extract rich in polyphenols maintains plasma lipid levels in hypercholesterolemic mice [26]; whereas the use of a mixture of resveratrol, CA and catechin significantly reduces the atheroma plaque in ApoE knockout mice [27]. Although the consumption of polyphenols contributes to the prevention of diseases, its oral administration without compound protection translates into a low efficiency at the action site. This is due to several factors such as concentration, binding site, chemical structure, stability in the gastrointestinal environment and aqueous solubility, which, in general, have a negative impact on absorption levels, metabolization degree, distribution throughout the body, life span and compound excretion [2,28]. Finally, the pharmacokinetics of polyphenols is also influenced by age, health status, intestinal microbiota and diet of patients, as well as by their oral antibiotic treatments [29,30]. All of the above is translated into different reports of low bioavailability of polyphenols, for example, 0.56–4.54 nmol/L for anthocyanins [31], 0.46–1.28 μmol/L for flavonones [32], and 37–60 nmol/L for phenolic acids [33].

Due to the low bioavailability of polyphenols after oral intake, several strategies have been developed to improve the bioavailability and bioactivity of these compounds. One of them is the use of microparticles based on biomaterials whose main function is to protect and transport the entire biomolecule [34]. Concerning this, nanoporous silicon (nPSi), is an excellent biomaterial that has been successfully used for the controlled release of different drugs and biomolecules, due to its large surface area, porous structure, biocompatibility, biodegradability, bioresorbability and resistance to low pH [35,36,37]. Moreover, because of the versatility of its surface chemistry, different functionalization strategies routes have been explored in order to enhance the load and controlled release of drugs [38]. A refined technique is to embed polymers into their nanopores to form composites [39]. In this regard, β-cyclodextrin (βCD), which is a biocompatible and aqueous soluble molecule, has been successfully used in drug delivery applications. The wide application of βCD in this field is related to the possibility to form the “host-guest” complexation (βCD/drug) [40]; drugs are encapsulated into its lipophilic cavity structure, whereas its outer hydrophilic surface can be crosslinked with other molecules (i.e., citric acid), yielding a 3D-polymer network suitable for drug delivery applications. Therefore by combining a flexible and soft βCD polymer within the highly porous inorganic matrix of nPSi as substrate, both stability and control of drugs release can be improved, increasing their therapeutic potential by reducing their degradation before they reach the target tissues [40]. Based on this, we hypothesize that nPSi-βCD composite is a safe alternative system for oral administration of CA and Pin since it has no toxic effects on human cells. The aim of this work is to determine if nPSi-βCD microparticles are a suitable and safe material for the load and controlled release of caffeic acid (CA) and pinocembrin (Pin), two of the main components of a Chilean propolis with anti-atherogenic and anti-angiogenic activity. This study includes the synthesis and physicochemical characterizations of nPSi-βCD microparticles loaded or not with CA or Pin, their respective release profiles and the corresponding cytocompatibility tests for each polyphenol and composite.

## 2. Materials and Methods

### 2.1. Materials

Caffeic acid (CA, *M*_W_ ≈ 180.16 g/mol), pinocembrin (Pin, *M*_W_ ≈ 256.25 g/mol), chitosan (Chi, 75–85% deacetylated, low *M*_W_ ≈ 5 × 10^4^ g/mol), β-cyclodextrin (βCD, *M*_W_ ≈ 1134.98 g/mol), citric acid (*M*_W_ ≈ 210.14 g/mol), g NaH_2_PO_2_·H_2_O (*M*_W_ ≈ 105.99 g/mol) and phosphate buffer solution (PBS) 0.01 M (0.138 M NaCl, 0.0027 M KCl, pH = 7.4 at 25 °C) were purchased from MiliporeSigma, St. Louis, MO, USA. Acetone (C_3_H_6_O), dimethyl sulfoxide (DMSO, C_2_H_6_OS), isopropanol (C_3_H_7_OH), ethanol (EtOH, C_2_H_5_OH), glacial acetic acid (CH_3_COOH), hydrogen peroxide (H_2_O_2_), sodium hydroxide (NaOH), hydrochloric acid (HCl) and hydrofluoric acid (HF) were acquired from Merck, Darmstadt, Germany. All chemicals were used without further purification, and solutions were prepared using Milli-Q water with resistivity of 18.2 M·Ω·cm (pH ∼7.6, otherwise mentioned). Silicon (Si) wafers (p^+^ type, boron-doped, orientation <100> resistivity of 0.001–0.005 Ω·cm) were purchased from University Wafer, South Boston, MA, USA. Fetal bovine serum (FBS), l-Glutamine, penicillin-streptomycin solution and D-PBS were purchased from Corning, Manassas, VA, USA. CellTiter-FluorTM assay and the CellTiter 96^®^ AQueous One Solution cell proliferation assay (MTS) were acquired from Promega, Madison, WI, USA.

### 2.2. Sample Preparation

**Figure 1:**
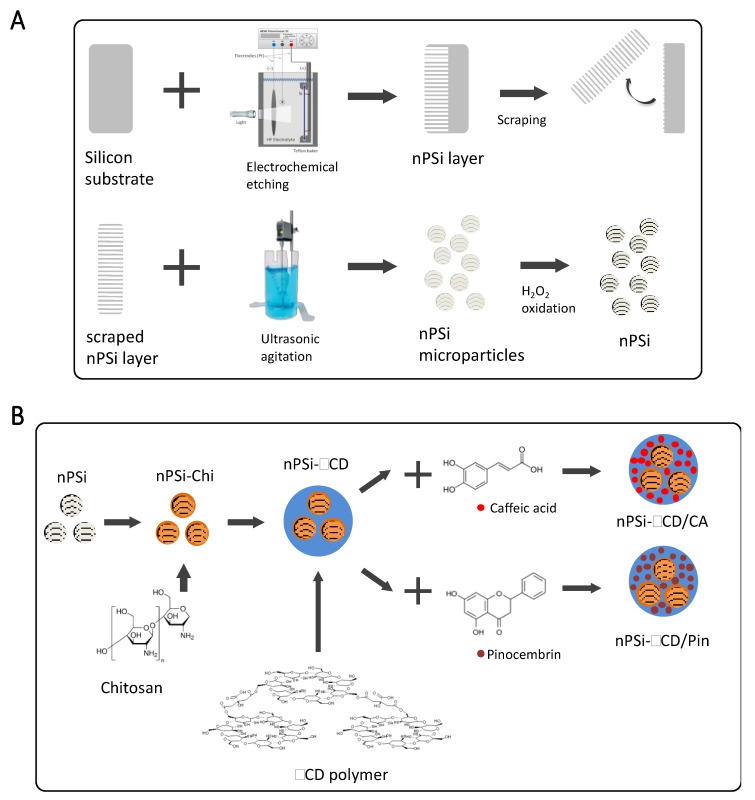
Experimental scheme for the synthesis of: (**A**) nPSi microparticles, and (**B**) nPSi-βCD composite microparticles. nPSi: nanoporous silicon, βCD: β-ciclodextrin polymer.

Si wafers were cleaned by ultrasonication in acetone, isopropanol and distilled water, for a period of 15 min in each solvent. Acetone removed greasy and oily substances; isopropanol was necessary to rinse acetone off, and distilled water removed any isopropanol residues. Then, nPSi layers were fabricated by electrochemical etching from the cleaned Si wafers in HF (48%):EtOH (1:2) solution under controlled formation conditions: etching time of 30 min and current density of 80 mA·cm^−2^. Afterward, an electropolishing pulse was applied to get free-standing nPSi layers. For that, the applied current density was enhanced to 150 mA/cm^2^ during 2 s. nPSi free-standing layers were scraped with a diamond tip to obtain microparticles. They were milled, collected in EtOH and subjected to 10 min ultrasound agitation for homogenization. Finally, the obtained nPSi particles were chemically oxidized by H_2_O_2_ (30%, *v*/*v*) for 12 h in orbital agitation and rinsed with EtOH (Figure 1A).

Oxidized nPSi microparticles were the substrate to synthetize the composite according to the protocol of Hernandez-Montelongo et al. [41] (Figure 1B). nPSi microparticles were immersed in a Chi solution for 15 min and after rinsed with EtOH (nPSi-CHI). The Chi solution (1% *w*/*v*) was previously prepared with Chi powder in 100 mM glacial acetic acid, then, the pH value was adjusted at 4 with a 0.1 M HCl and/or NaOH solution. For the composites (nPSi-βCD) synthesis, a monomer solution was prepared with 10 g βCD, 3 g NaH_2_PO_2_·H_2_O as catalyst, and 10 g citric acid in 100 mL of distilled water. Then, nPSi-Chi was immersed in this solution for 15 min while stirring. Samples were dried, first at room temperature, and later at 90 °C for 1 h in each case. The βCD–citric acid in situ polymerization in nPSi-CHI was carried out at 140 °C for 25 min. Afterward, samples were rinsed with EtOH, dried at 90 °C for 1 h and milled for homogenization.

### 2.3. Physicochemical Characterization

The zeta potential of samples was measured by a ZetaSizer Nano–ZS (Malvern Ltd., Royston, UK) in distilled water. Attenuated total reflectance Fourier-transform infrared spectroscopy (ATR-FTIR) was used for chemical analyses of the microparticles. An FTIR spectrometer (CARY 630 FTIR Agilent Technologies, Santa Clara, CA, USA) was used in a range between 4000 and 600 cm^−1^ with a resolution of 1 cm^−1^ (NS = 4). The obtained spectra were mathematically processed by data smoothing and spectral normalized. The morphology of the samples was investigated by a variable pressure scanning electron microscope (VP-SEM, SU-3500 Hitachi, Tokyo, Japan) using an acceleration voltage of 5 kV. The size distribution of samples was presented as histograms; data was obtained from the SEM images that were processed using freely available ImageJ software, version 1.52k, National Institutes of Health, Bethesda, Maryland, USA. The atomic percentage was obtained by energy-dispersive X-ray analysis (EDX) with an INCA X-sight from Oxford Instruments within the VP-SEM equipment. Thermogravimetric analyses (TGA) were conducted in a N_2_ atmosphere at a heating rate of 10 °C/min (DTG-60H Shimadzu, Tokyo, Japan). Porosity of nPSi samples was obtained by gravimetric analysis according to the following equation:

*%P* = (*m_1_ − m_2_/m_1_ − m_3_*) × 100 (1)

where *m_1_* is the mass of Si wafer before electrochemical etching, *m_2_* is the mass of sample just after anodization and *m_3_* is the mass of sample after a rapid dissolution of the whole porous layer in a 3% KOH solution.

### 2.4. Polyphenols Loading

Polyphenols, CA and Pin, were reconstituted with 100% DMSO (200 μM) and stored at −20 °C until required. Five mg of samples were loaded with CA and Pin using 1 mL of concentrated aqueous solution of each polyphenol (2 mM) and placed in a horizontal shaker incubator (NB-2005LN Biotek, Winooski, VT, USA) for 12 h at 50 RPM and room temperature. After polyphenol loading, samples were rinsed to remove the unentrapped molecules, they were dried at room temperature and milled for homogenization. To determine the maximum polyphenol loading, samples were hydrolyzed in 0.1 M NaOH solutions then they were analyzed by UV–visible spectrometry (UVmini-1240 spectrometer Shimadzu, Tokyo, Japan). CA and Pin were detected at 310 and 322 nm, respectively. Polyphenol entrapment efficiency (%PEE) and polyphenol loading efficiency (%PLE) and were calculated from Equations (2) and (3), respectively [42]:

*%PEE* = (*m_p_m_/m_p_i_*) × 100 (2)

*%PLE* = (*m_p_m_/m_m_*) × 100 (3)

where *m_p_m_* is the mass of polyphenol in microparticles, *m_p_i_* is the mass of polyphenol fed initially and *m_m_* is the mass of microparticles.

### 2.5. Polyphenols Release Profiles

Polyphenols release data were collected at different times using 5 mg of charged samples in 3 mL of PBS solution (37 °C) as release medium in agitation at 100 RPM. All experiments were conducted in triplicate and nPSi samples were used as controls in these kinetic experiments.

In order to determine the mechanism of drug release, three models were fitted to the release profiles: First order, Higuchi and Korsmeyer–Peppas models. The first order equation is [43]:

*ln M_t_ − ln M*_0_*= k*_1_*t* (4)

where *M_t_* is the absolute cumulative amount of drug released at time point *t*, *M*_0_ is the initial amount of drug in the solution, and *k_1_* is the first order release kinetic constant. The Higuchi equation is [44]:

*M_t_ = k_H_t*^1/2^ (5)

where *M_t_* is the absolute cumulative amount of drug released at time point *t*, and *k_H_* is Higuchi release kinetic constant. The Korsmeyer–Peppas semiempirical model is given by [45]:

*M_t_/M_∞_ = k_KP_t^n^* (6)

where *M_t_/M_∞_* is the fractional drug release, *t* is the release time, *k_KP_* is the Korsmeyer–Peppas release kinetic constant and *n* is an exponent which characterizes the mechanism of release. The fitting of models was conducted with SigmaPlot v14.0, Systat Software, Inc., San Jose, USA.

### 2.6. Cytotoxicity Assays

#### 2.6.1. Cell Culture

For cell culture, human umbilical vein endothelial cells (HUVECs) were obtained from the Cell Applications Inc (San Diego, CA, USA), and maintained in Endothelial Cell Growth Medium (Cell Applications, San Diego, CA, USA) supplement with 10% FBS, 1% l-Glutamine and 1% penicillin-streptomycin solution. The cell culture was routinely grown under specific conditions in a humidified atmosphere incubator of 95% air and 5% CO_2_ at 37 °C. Cells were used at no more than seven passages.

#### 2.6.2. Polyphenols Cytotoxicity

For the in vitro viability assays, CellTiter 96^®^ Aqueous One Solution Cell Proliferation Assay (MTS) Promega (Madison, WI, USA) was used to determine the toxic effect of CA and Pin on HUVECs viability. The MTS assay is based on the conversion of a tetrazolium salt into a colored aqueous soluble formazan product by mitochondrial activity of viable cells at 37 °C. The amount of formazan produced by dehydrogenase enzymes is directly proportional to the number of living cells in culture. The viability assays were performed according to the manufacturer’s protocols. HUVECs were briefly placed into 96-well plates (2.5 × 10^3^ cells/per well) in 100 μL and incubated at 37 °C. Then, cells were exposed to increase concentrations up to 2000 μM of polyphenols. The compound was prepared in dimethylsulfoxide (DMSO). After 24 h of incubation, the medium was removed and 20 μL MTS reagent was added to the wells, followed by a 4-h incubation at 37 °C. The absorbance was determined by a microplate reader (NanoQuant, Infinite^®^ M200PRO–Tecan, Redwood, CA, USA) at 490 nm. Results were expressed as the percentage of viability relative to the control. The cell viability was calculated as follows: cell viability (%) = (OD of treatment group/OD of control group) × 100. Dose-dependent viability curves were determined using the cell viability trends.

#### 2.6.3. nPSi-βCD Composite Cytotoxicity

To determine the effect of the composite (nPSi-βCD) on HUVEC cell viability, a CellTiter-Fluor^TM^ Cell Viability assay (Promega, Madison, WI, USA) was used. This assay measures a conserved and constitutive protease activity within live cells using a fluorogenic peptide substrate (glycyl-phenylalanyl-aminofluorocoumarin; GF-AFC). The substrate enters intact cells where it is cleaved by the live-cell protease activity to generate a fluorescent signal proportional to the number of living cells. 1 × 10^5^ cells were exposed to different concentrations of composite and were photographed using a confocal laser microscope (CLSM, FV1000 Olympus, Tokyo, Japan) with excitation and emission wavelengths of 390 nm and 505 nm, respectively. The assay was performed according to the manufacturer’s protocols. The fluorescence intensity analysis was performed with Olympus Fluoview (FV10 v2.0c) software (Olympus Corporation, Tokyo, Japan). Data was analyzed statistically by analysis of variance (ANOVA) using Kruskal-Wallis test, and post-hoc test were also conducted using Dunn’s multiple comparisons. The level of significance was *p* < 0.05 and the results were expressed as the arithmetic mean of three biological replicates with its corresponding standard deviation. The statistical analysis was performed using the GraphPad Prism v7.0c (GraphPad Software, San Diego, CA, USA).

## 3. Results and Discussion

**Figure 2:**
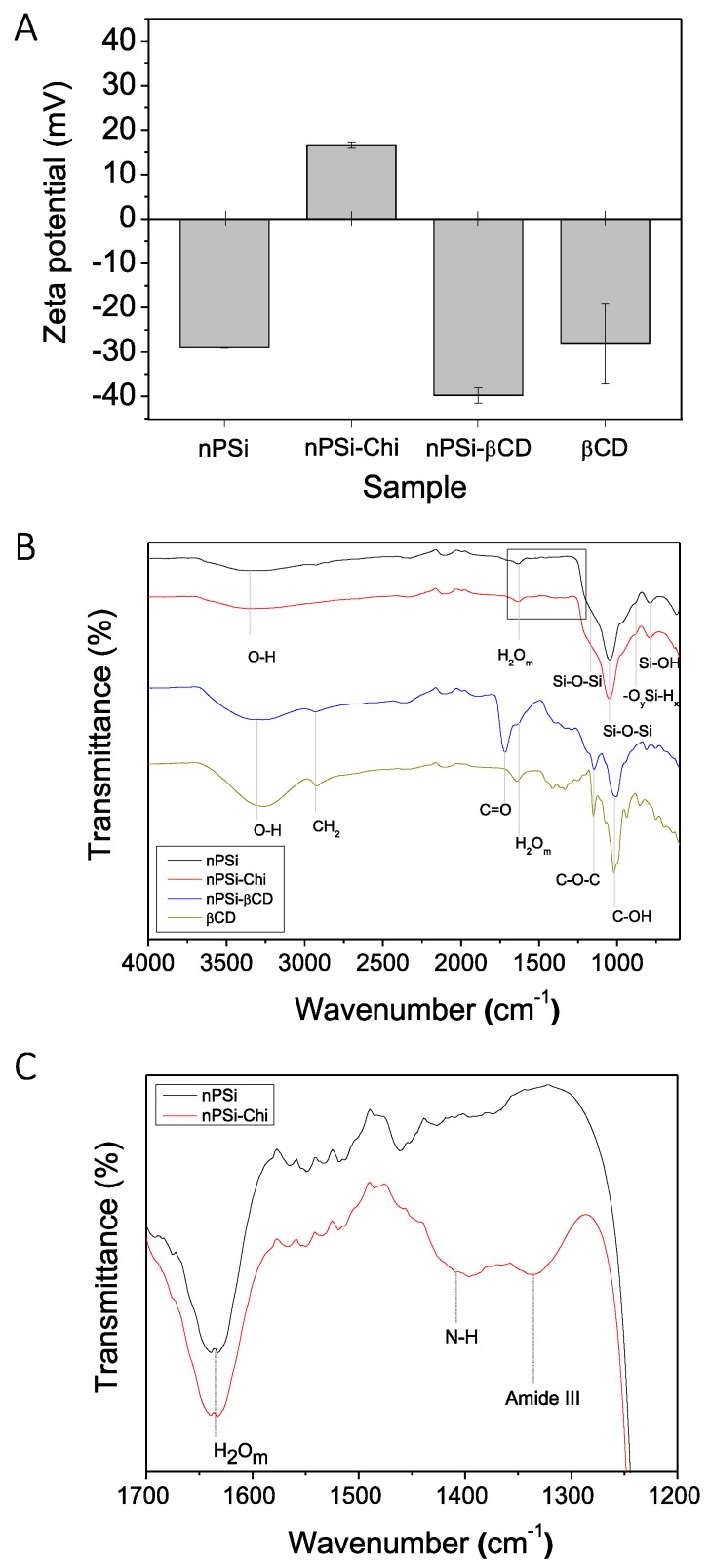
Monitoring of the synthesis process of nPSi-βCD composite microparticles via: (**A**) Zeta potential and (**B**) Attenuated total reflectance Fourier transform infrared spectroscopy analysis (ATR-FTIR) (**C**) Zoom in of ATR-FTIR spectra.

As the synthesis of composite microparticles was obtained by electrostatic attraction of oppositely charges, zeta potential analysis was performed (Figure 2A). This technique provides the net electrical charge of the microparticles generated by their functional groups. In the case of nPSi, its negative zeta potential value (−29.06 ± 0.06 mV) would correspond to the negatively charged silanol groups produced by the chemical oxidation with H_2_O_2_ [46]. nPSi-Chi showed positive values (16.5 ± 0.6 mV) because the grafting with chitosan would generate a rich aminated surface [47]. On the other hand, the sharp negative zeta potential of nPSi-βCD (−39.8 ± 1.73 mV) was according to βCD value (−28.2 ± 9 mV), which is generated by the hydrophilic outer surface cavity (C–OH groups) of βCD molecules [48].

In that sense, ATR-FTIR analysis was performed to determine the chemical changes of nPSi microparticles during the cascade synthesis processes (Figure 2B). The spectrum of nPSi showed a sharp transmittance peak at 1050 cm^−1^ with a shoulder at 1170 cm^−1^, which both correspond to Si–O–Si stretching mode [49]. Besides, weak bands at 880 and 795 cm^−1^ related to −O_y_Si-H_x_ and SiOH, respectively, and the O–H stretching band from SiOH and adsorbed H_2_O at 3350 cm^−1^ were detected [49]. Moreover, molecular water (H_2_O_m_) absorbance band was observed at 1630 cm^−1^ [50]. These detected functional groups are in agreement with the chemical oxidation of nPSi via H_2_O_2_. On the other hand, the spectrum of nPSi-Chi presented the same functional groups as nPSi plus weak bands of N–H and amide III detected at 1408 and 1320–1346 cm^−1^ [51,52,53], respectively (Figure 2C). Those bands are related to the polyamino-saccharide chains of Chi, which were used to link the βCD polymer with nPSi microparticles. Regarding the spectrum of nPSi-βCD, bands corresponded to the spectrum of native βCD were observed: C–OH stretching (1021 cm^−1^) [49], C–O–C stretching (1150 cm^−1^) [13], H_2_O_m_ (1630 cm^−1^) [50], CH2 asymmetric stretching (2930 cm^-1^) and O-H stretching from hydroxyl groups (3300 cm^−1^) [49]. nPSi-βCD barely showed an extra band than βCD at 1721 cm^−1^ which correspond to C=O groups generated during the polymerization achieved between βCD and citric acid [49].

**Figure 3:**
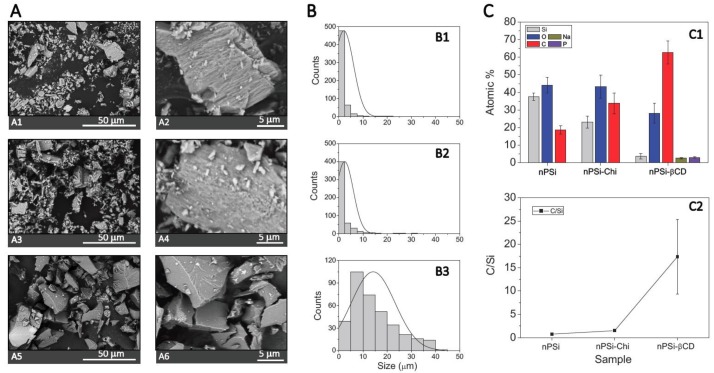
(**A**) Scanning electron microscope images (SEM) of samples at the different stages of synthesis: (**A1**,**A2**) for nPSi, (**A3**,**A4**) for nPSi-Chi, (**A5**,**A6**) for nPSi-βCD. (**B**) Histograms of particle size distribution: (**B1**) for nPSi, (**B2**) for nPSi-Chi, (**B3**) for nPSi-βCD. (**C**) Atomic % and C/Si ratio of samples obtained from energy-dispersive X-ray analysis (EDX), (**C1**,**C2**), respectively.

SEM images were produced (Figure 3A) to analyze the size and morphology of samples at the different stages of synthesis. Moreover, the obtained distribution size from these images is shown in Figure 3B. nPSi and nPSi-Chi presented irregular shapes with an average size of 2.0–2.5 μm, and both kind of microparticles showed rougher surface due to their columnar pores of ~50 nm width. In addition, gravimetric analysis presented an average porosity of 75 ± 5%. In the case of the nPSi-βCD sample, the microparticle shapes were also irregular with a higher size around of 14.0 μm, and their faces exhibited a softer appearance. In fact, folds produced by the polymerization could be also observed. The increase in particle size may have been because the small particles agglomerated during the polymerization forming higher particles. Similar size distribution of this kind of particles for oral drug delivery system has been previously reported by Salonen et al. [54]. On the other hand, EDX analysis was performed on each sample (Figure 3C1) and the atomic percentage and C/Si ratio were obtained (Figure 3C2). nPSi mainly exhibited Si and O signal due to the oxidation performed by H_2_O_2_. Although the C/Si ratio was 0.76 ± 0.2, C signal was considerable high (~18%). This was most probably due to contamination when handling. In the case of nPSi-Chi, due to the previous characterization (Zeta potential and ATR-FTIR), N signal from amines groups of the incorporated chitosan was expected to be identified but it was not. This can be explained because incorporated chitosan was most likely a superficial layer and N signal was not strong enough to be detected by EDX technique. However, it is possible to observe that the C/Si ratio increased twice up to 1.5 ± 0.3 due to the polymer grafting [55]. Regarding the nPSi-βCD sample, a high increase of C signal was identified: the C/Si was raised up to 17.4 ± 8 due to the in situ polymerization of βCD and citric acid. In fact, Na and P traces from the catalyst were also detected.

**Figure 4:**
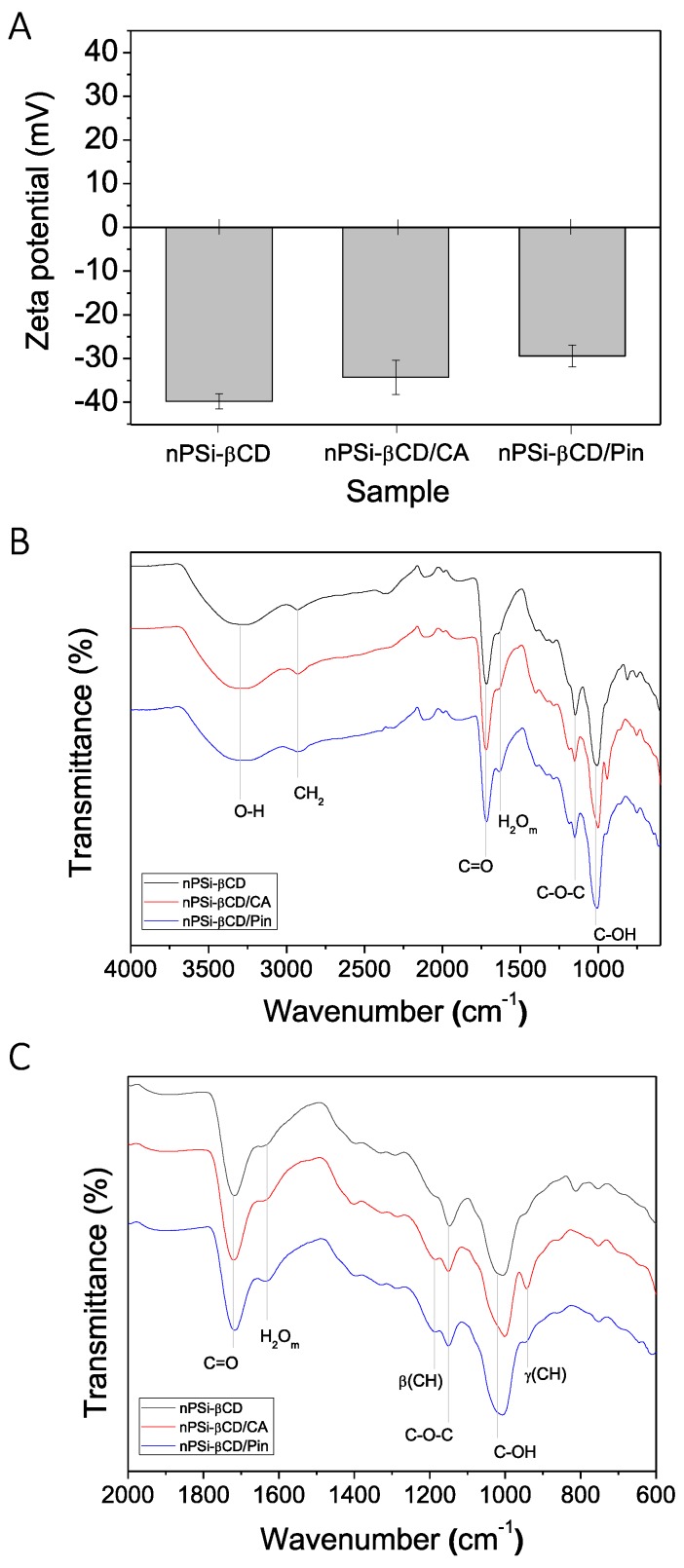
Monitoring of the polyphenols loading on PSi-βCD composite microparticles via: (**A**) Zeta potential and (**B**) Attenuated total reflectance Fourier transform infrared spectroscopy analysis (ATR-FTIR). (**C**) Zoom in of ATR-FTIR spectra.

In order to evaluate changes in nPSi-βCD composite microparticles with the loaded polyphenols, same set of previous physicochemical characterization was carried out. Figure 4A shows how zeta potential of nPSi-βCD was reduced after the addition of both polyphenols: nPSi-βCD/CA and nPSi-βCD/Pin presented 5.5 and 10.4 mV lower zeta potential values than nPSi-βCD, respectively. Regarding the ATR-FTIR analysis (Figure 4B), nPSi-βCD/CA and nPSi-βCD/Pin spectra exhibited the same functional groups than nPSi-βCD spectrum. Nevertheless, both nPSi-βCD/CA and nPSi-βCD/Pin exhibited two extra bands related to the bending modes of CH, which are associated to incorporation of both polyphenols (Figure 4C): β(CH) and γ(CH) at 1187 and 940 cm^−1^, respectively. β denotes in-plane bending modes and γ designates out-of-plane bending modes [56].

**Figure 5:**
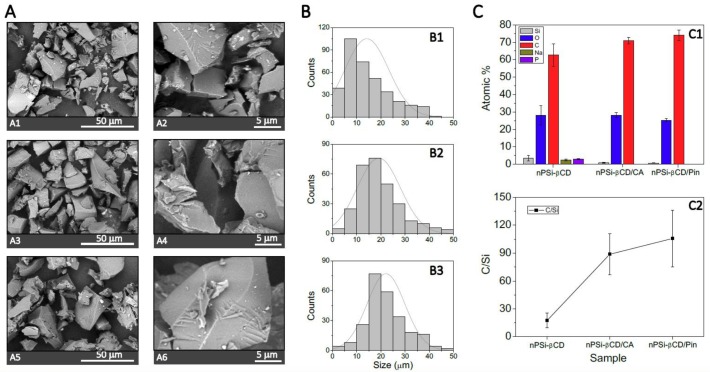
(**A**) Scanning electron microscope images (SEM) of nPSi-βCD loading with polyphenols: (**A1**,**A2**) for nPSi-βCD, (**A3**,**A4**) for nPSi-βCD/CA, (**A5**,**A6**) for nPSi-βCD/Pin. (**B**) Histograms of particle size distribution: (**B1**) for nPSi-βCD, (**B2**) for nPSi-βCD/CA, B3 for nPSi-βCD/Pin. (**C**) Atomic % and C/Si ratio of samples obtained from energy-dispersive X-ray analysis (EDX), (**C1**,**C2**), respectively.

Regarding morphology, which was observed by SEM images (Figure 5A), nPSi-βCD/CA and nPSi-βCD/Pin did not present significantly changes in comparison with nPSi-βCD; the surface of both nPSi-βCD/CA and nPSi-βCD/Pin microparticles exhibited a softer appearance and some folds produced by the polymerization. However, the size of the loaded microparticles was higher than nPSi-βCD (Figure 5B): ~19 and ~22 μm, respectively. As polyphenols are highly hydrophobics, they could tend to agglomerate smaller particles. As Pin is more hydrophobic than CA, this could generate more aggregation, and therefore, higher microparticles. On the other hand, EDX analysis were also performed on the samples (Figure 5C1), atomic percentage and C/Si ratio were obtained too (Figure 5C2). nPSi-βCD/CA and nPSi-βCD/Pin did not present any more Na and P traces, and the C/Si ratio considerably increased in comparison with nPSi-βCD: nPSi-βCD showed a C/Si ratio of 17.4 ± 8, and nPSi-βCD/CA and nPSi-βCD/Pin presented 88.75 ± 22.2 and 105.7 ± 30.5, respectively.

**Figure 6:**
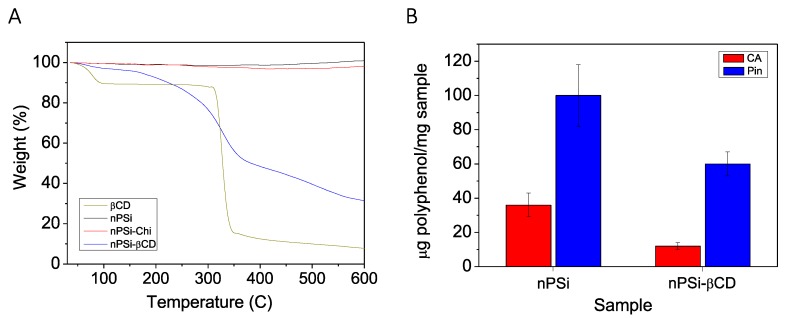
(**A**) Thermogravimetric analysis (TGA) of microparticles at different stages of synthesis, and (**B**) Polyphenols capacity loading of nPSi-βCD composite microparticles.

In order to determine the functionalization degree of Chi and βCD polymer integrated onto nPSi substrates, TGA analyses were performed (Figure 6A). The plot illustrates the percent mass as a function of samples temperature under a nitrogen purge. As expected, nPSi sample practically did not present degradation, but nPSi-Chi showed a slight decomposition of around 3%, this is in accordance previous characterization that suggests chitosan grafting was just superficially. Moreover, the thermogravimetric analysis of native βCD was monitored as reference. The βCD decomposition was clearly appreciable; the first stage with was at 100 °C corresponds to the level of absorbed water (~10.5%). The second stage, which started at 310 °C and finished at 350 °C, is related to the melting, decomposition and turning into char of the glucose units of the βCD molecules [57]. In the case of nPSi-βCD, the phenomenon was gradual, due to the stronger 3D structure net of βCD polymer, but similar to the native βCD reference. Considering the residual weight at 600 °C, it is possible to ponder that nPSi-βCD was composed by 32% nPSi, 62% βCD polymer, 3% Chi and 3% humidity. The high percent of βCD polymer (62%) in composite composition can be explained, in addition to the electrostatic interactions with polymers showed by zeta potential, with porosity of samples which also worked as an anchor holding the polymer film.

%PEE and %PLE of samples were determined by UV-Vis spectroscopy at 310 and 322 nm for CA and Pin, respectively. Although nPSi (control) did not exhibit a chemical surface compatible with polyphenols, samples presented high values of %PEE 50 ± 2.0 and 97.5 ± 2.0 for CA and Pin respectively. This can be explained by the high surface area of their nanopores. In the case of nPSi-βCD, microparticles exhibited 16.6 ± 1.0 %PEE and 58.5 ± 1.5 %PEE for CA and Pin, respectively. In the same sense, nPSi samples presented higher %PLE than nPSi-βCD. Figure 6B shows the polyphenols capacity loading of both kind of microparticles. nPSi presented a load of 36 ± 7 µg CA/mg nPSi (3.6 ± 0.7% PLE) and 100 ± 18 µg Pin/mg nPSi (10.0 ± 1.8% PLE), and nPSi-βCD showed a load of 12 ± 2 µg CA/mg nPSi (1.2 ± 0.2% PLE) and 60 ± 7 µg Pin/mg nPSi (6.0 ± 0.7 %PLE). Due to previous characterization results, it is very possible that polyphenols were mainly adsorbed in the large corona of βCD polymer around the small nPSi microparticles, which were the substrate of the composite.

**Figure 7:**
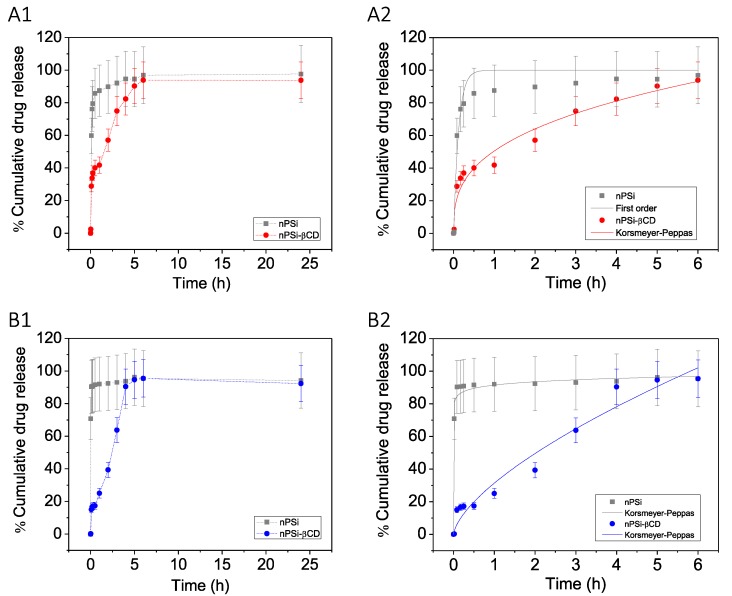
Polyphenols release profiles in phosphate-buffered saline (PBS) at 37 °C: (**A1**,**A2**) CA and (**B1**,**B2**) Pin.

To evaluate the polyphenols controlled release functionality, loaded microparticles of nPSi and nPSi-βCD were immersed in PBS batches at 37 °C under stirring. The obtained polyphenols release profiles are shown in Figure 7A1,A2 for CA, and Figure 7B1,B2 for Pin. After 24 h of release, all samples presented higher values of %cumulative release. nPSi samples showed 97.6 ± 17.6 and 94.2 ± 17.0 for CA and Pin, respectively. In the case of nPSi-βCD, microparticles exhibited 93.8 ± 11.2 and 92.3 ± 11.0 for CA and Pin, respectively. Profiles presented a clear contrasting behavior between the control (nPSi) and composite (nPSi-βCD). Results visibly showed that nPSi-βCD worked much better than nPSi: both polyphenols retained into nPSi showed a fast release profile during the first minutes, in contrast with nPSi-βCD, which showed a controlled released for more than 5 h.

To attain deeper perception of the mechanisms that govern the release of polyphenols from the samples, three release models were fitted to the experimental data: first order, Higuchi and Korsmeyer-Peppas models (Table 1). In the case of CA release, according to the *r^2^* obtained values for nPSi, it presented better adjustment with the first order model, where immediate-release dosage was dispersed in a single action [58]. However, for the release of CA using nPSi-βCD, CA release kinetics were described with a more accurate precision by the Korsmeyer-Peppas model. This means that the governing factor of CA release was not the dissolution from samples, but a Fickian diffusion process [41]. Moreover, in that sense, since the release exponent *n* from the Korsmeyer-Peppas model was smaller than 0.5, only diffusive release can be suggested. Therefore, erosion process could be insignificant [41]. For the case of Pin, both release profiles from nPSi and nPSi-βCD microparticles, were better adjusted to the Korsmeyer-Peppas model. However, nPSi presented an *n* value closed to zero. Regarding nPSi-βCD, it showed an *n* > 0.5, which suggest that besides Fickian diffusion, erosion process could also be contributing in the Pin release [41].

**Figure 8:**
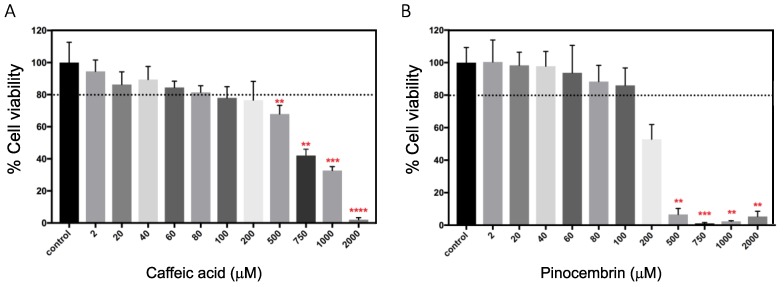
Viability of HUVECs treated with polyphenols. Cells were exposed to different concentrations of polyphenols for 24 h and cellular viability was measured by tetrazolium salt (MTS) assay. (**A**) Cells treated with 2–2000 mM of caffeic acid, and (**B**) Cells treated with 2–2000 mM of pinocembrin. Unexposed cells to nPSi-βCD microparticles were used as a control. The dashed line indicates the cell viability of 80%. All results are presented as the mean ± standard deviation (SD). The experimental data from all relevant studies were analyzed using Analysis of Variance (ANOVA) and Kruskal–Wallis test, which indicate the statistical significance when the percentage of cells viability exposed to the different microparticle concentrations are different from the control. ** *p* < 0.01, *** *p* < 0.001, **** *p* < 0.0001, (n = 3).

To evaluate the cytotoxic effect of CA and Pin, human umbilical vein endothelial cells (HUVECs) cultures were performed. HUVECs are a classic model to study endothelial functions, such as angiogenesis. Angiogenesis is the formation of new blood vessels from pre-existing vessels [59]. Although it is a physiological process, the abnormal growth of vessels promotes the development and/or progression of some diseases such as cardiovascular diseases. Regarding this, the MTS test was used to study the impact of CA and Pin on the viability of HUVECs. Results showed that viability gradually decreased and responded in a dose-dependent manner for both polyphenols. In the case of CA, cell viability was slightly reduced from 100% to 80% for concentrations from 2 to 200 μM (*p* > 0.05), while surviving cells were ≤70% for concentrations ≥500 μM. (*p* < 0.01) (Figure 8A). Regarding to the effect of Pin (Figure 8B), the viability was higher than 80% from 2 to 100 μM, but it decreased to 50% at 200 μM (*p* > 0.05). Moreover, cell viability was reduced to less than 20% at concentrations ≥ 500 μM (*p* < 0.01). According to this, concentrations up to 200 and 100 μM for CA and Pin, respectively, maintained cell viability ≥80%, that is to say, they did not generate cellular cytotoxicity.

**Figure 9:**
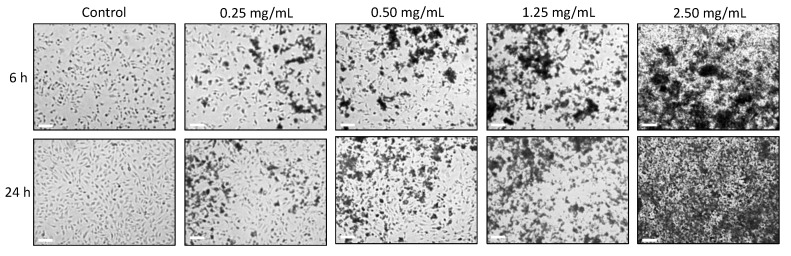
Microscopical images of the effect of nPSi-βCD microparticles exposition to HUVECs during a 6 and 24 h culture. Images were taken at 8X magnification. Scale bars: 1 mm.

On the other hand, the effect nPSi-βCD microparticles on the viability of HUVECs was also studied. Cells were cultured in the presence of composite microparticles at concentrations of 0.25, 0.50, 1.25 and 2.5 mg/mL for 6 and 24 h. In the microscopic observation (Figure 9), HUVECs exposed to the lowest concentrations (0.25 and 0.50 mg/mL) exhibited a normal flattened and thin morphology, suggesting that microparticles were well tolerated by cells. Instead, the highest concentrations (1.25 and 2.5 mg/mL), generated a large amount of rounded and suspended cells in the culture medium. This indicates that nPSi-βCD microparticles concentrations higher than 0.50 mg/mL were not well tolerated by HUVECs, affecting its cell adhesion capacity, an essential survival characteristic of this type of cells.

**Figure 10:**
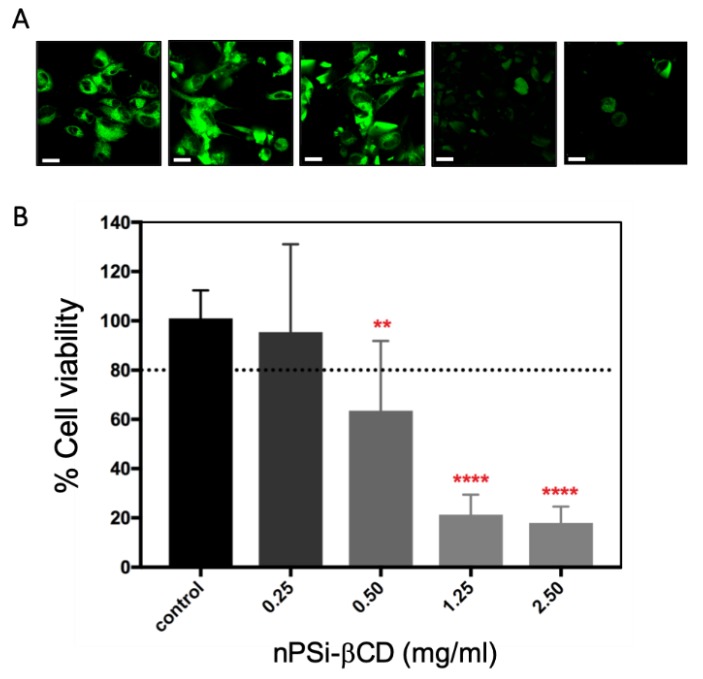
Cytotoxicity of HUVECs exposed to nPSi-βCD microparticles. The cells were exposed for 6 h and then cellular viability was evaluated with CellTiter-FluorTM assay and fluorescence intensity measuring was with confocal laser microscope (**A**) HUVECs exposed to nPSi-βCD microparticles. Scale bars: 20 μm. (**B**) Viability percentage of cells exposed to composite microparticles. Unexposed cells to nPSi-βCD microparticles were used as a control. Dashed line indicates cell viability of 80%. All results are presented as the mean ± standard deviation (SD). The experimental data from all the studies were analyzed using Analysis of Variance (ANOVA) and Kruskal–Wallis test, which indicate the statistical significance when the percentage of cells viability exposed to the different microparticles concentrations are different from the control. ** *p* < 0.01, **** *p* < 0.0001. (n = 3).

Concerning the viability percentage of HUVECs exposed to different concentrations of nPSi-βCD microparticles (Figure 10B), results showed that cell viability at 0.25 mg/mL was higher than 80% but it started to decrease at concentrations equal or higher than 0.50 mg/mL (63%; *p* < 0.01). Specifically, at concentrations of 1.25 and 2.50 mg/mL, cell viability was very low reaching values as low as 20%. This increase in cell mortality from 0.25 mg/mL to ≥1.25 mg/mL of nPSi-βCD microparticles may be due to an alteration in the basic cellular functions such as the adherence capacity affected, for example, cell communication, differentiation and migration leading to cell death [60].

## 4. Conclusions

Physicochemical characterizations showed that nPSi-βCD microparticles were suitable to be used an alternative as carrier and controlled oral delivery system of both polyphenols, CA and Pin. The release profiles indicated that nPSi-βCD composite presented a better-controlled release of polyphenols than nPSi without βCD polymer. Moreover, nPSi-βCD samples loaded higher amount of Pin than CA, and the release of Pin was higher controlled than CA. For the CA case, a purely diffusive mechanism of release was suggested, but for the Pin, erosion process could be also contributing during the release. On the other hand, nPSi-βCD microparticles presented cytocompatibility HUVECs culture at concentrations of 0.25 mg/mL. Then, these results indicate that nPSi-βCD composite microparticles could be safely used as an alternative oral delivery system to improve controlled release and bioavailability of CA and Pin, and eventually other polyphenols with therapeutic potential.

## Figures and Tables

**Table 1:** *In vitro* release kinetics of caffeic acid and pinocembrin in PBS at 37 °C.

**Polyphenol**	**Sample**	**First order**		**Higuchi**		**Korsmeyer-Peppas**		
		$lnM_{t}-lnM_{0}=k_{1}t$		$M_{t}=k_{H}t^{1/2}$		$\frac{M_{t}}{M_{\infty }}=k_{KP}t^{n}$		
		$k_{1}\left(\%h^{-1}\right)$	$r_{adj}^{2}$	$k_{H}\left(\%h^{-1/2}\right)$	$r_{adj}^{2}$	$k_{KP}\left(h^{-n}\right)$	n	$r_{adj}^{2}$
Caffeic acid	nPSi	8.1047	0.9355	52.7074	−0.0040	79.7830	0.197	0.7832
	nPSi-βCD	0.5694	0.7982	41.6744	0.9074	50.4833	0.3419	0.9649
Pinocembrin	nPSi	76.3252	0.9271	54.5954	−2.1444	91.4797	0.0317	0.9774
	nPSi-βCD	0.3892	0.9433	38.5733	0.9451	31.3890	0.6584	0.9654
